# Cathepsin B Regulates Mice Granulosa Cells’ Apoptosis and Proliferation In Vitro

**DOI:** 10.3390/ijms222111827

**Published:** 2021-10-31

**Authors:** Chao Chen, Muhammad Jamil Ahmad, Tingzhu Ye, Chao Du, Xinxin Zhang, Aixin Liang, Liguo Yang

**Affiliations:** 1 Key Lab of Agricultural Animal Genetics, Breeding and Reproduction of Ministry of Education, College of Animal Science and Technology, Huazhong Agricultural University, Wuhan 430070, China; chenchao1995@webmail.hzau.edu.cn (C.C.); jameel_uaf@webmail.hzau.edu.cn (M.J.A.); yetingzhu@webmail.hzau.edu.cn (T.Y.); duxiaochaoshuai@gmail.com (C.D.); dameichaomei@gmail.com (X.Z.); lax.pipi@mail.hzau.edu.cn (A.L.); 2 Hubei Province’s Engineering Research Center in Buffalo Breeding and Products, Wuhan 430070, China

**Keywords:** *CTSB*, mice, proliferation, apoptosis, granulosa cells

## Abstract

Cathepsin B (*CTSB*), a lysosomal cysteine protease’s high expression and activity, has been reported to cause poor-quality embryos in porcine and bovine. Nevertheless, *CTSB* functions in mice granulosa cells remain to explore. To discuss the *CTSB* functional role in follicular dynamics, we studied apoptosis, proliferation, cell cycle progression, and related signaling pathways in primary mouse granulosa cells transfected with small interference RNA specific to *CTSB* (siCTSB) for 48 h. Further, mRNA and protein expression of cell proliferation regulators (*Myc* and *cyclin D2*), apoptosis regulators (*caspase 3*, *caspase 8*, *TNF-α*, and *Bcl2*), steroidogenesis-related genes (*FSHR* and *CYP11A1*), and autophagy markers (*LC3-I* and *ATG5*) were investigated. In addition, the effect of *CTSB* on steroidogenesis and autophagy was also examined. Flow cytometry analysis assay displayed that silencing of *CTSB* decreased the early and total apoptosis rate by downregulating *TNF-α*, *caspase 8*, and *caspase 3*, and upregulating *Bcl2*. By regulating *Myc* and *cyclin D2* expression and activating the p-Akt and p-ERK pathways, *CTSB* knockdown increased GC proliferation and number. A significant decline in estradiol and progesterone concentrations was observed parallel to a significant decrease in autophagy-related markers *LC3-I* and *ATG5* compared to the control group. Herein, we demonstrated that *CTSB* serves as a proapoptotic agent and plays a critical role in folliculogenesis in female mice by mediating apoptosis, autophagy, proliferation, and steroidogenesis. Hence, *CTSB* could be a potential prognostic agent for female infertility.

## 1. Introduction

Female reproduction pathways are the driving forces in evolution, including folliculogenesis, ovulation, fertilization, embryo development, parturition, and lactation [1]. The central functional units of the mammalian ovary, including granulosa cells (GCs) and theca cells (TCs), experience functional, morphological, and physiological changes during folliculogenesis; a spontaneous, most complex, and intricate reproductive phenomenon [2].

During the development of ovarian follicles, all stages of follicular atresia are associated with the apoptosis or death of granulosa cells (GCs). Based on this observation, GC apoptosis or death is considered the primary mechanism underlying follicular atresia [3,4]. In mammalian ovarian follicular development, only limited follicles reach ovulation, while the rest suffer from atresia at various stages of development. [5]. In parallel to apoptosis, nonapoptotic forms of programmed cell death, such as autophagy and necrotic-like cell death, have also been observed in the antral follicles of geese and quails [6]. Autophagy is an internal bulk degradation system in which autophagosomes, a part of the cytoplasm enclosed in double membrane-bound structures, mature and unite with lysosomes for degradation [7,8]. Autophagy promotes cell death by excessive self-digestion and degradation of essential cellular constituents [6], and various stimuli that induce apoptosis could trigger autophagy [9,10]. In humans, the exposure of an oxidized low-density lipoprotein has caused granulosa cells’ death by autophagy via inducing endothelial cell apoptosis [11]. Taken together, autophagy may be involved in folliculogenesis, as granulosa cells are the primary site of apoptosis during follicle atresia [12]. Previously, a study has demonstrated induction of autophagy in granulosa cells during folliculogenesis and a strong correlation with apoptosis in rat granulosa cells [6].

Despite overwhelming evidence for ovarian follicular atresia, the cellular and molecular mechanisms underlying this condition remain unknown. Therefore, it is imperative to comprehend dynamically regulated ovarian follicular growth, proliferation, and apoptosis. In addition, identifying novel regulatory molecules with an underlying GC function mechanism is critical to understand folliculogenesis thoroughly. A thorough understanding of folliculogenesis could aid in the development of novel molecular diagnostic and therapeutic approaches to combat the ever-increasing infertility problem in mammals.

Intracellular proteins are degraded in lysosomes by a lysosomal cysteine protease called *CTSB* [13], which controls various biological processes such as cell death, proliferation, migration, and cancer [14]. Before incorporating into the acidic lysosome environment, a *CTSB* (enzyme precursor) inactive form is converted into an active form through post-translational modifications [15,16]. *CTSB* active form has heavy and light-chain subunits linked by disulfide with a molecular weight of 30 kDa [17], which critically regulate various physiological and pathological processes, including initiating apoptosis and extracellular matrix remodeling. *CTSB*, either directly or indirectly, plays a critical role in activating the apoptotic pathway via initiator caspases rather than executioner caspases [18]. Indirect regulation of caspases by *CTSB* is mediated through mitochondrial membrane degradation, which translocates apoptosis-inducing components from the mitochondria to the cytoplasm [19].

Different cells have been reported to expressing the *CTSB*, such as cumulus–oocyte complex (COCs) and embryos in bovine and porcine [20,21,22]. *CTSB* activity and high protein levels were determined in poor-quality bovine and porcine embryos. In particular, inhibiting *CTSB* activity reversed these effects and improved preimplantation embryos in bovine and porcine models [20,23]. In addition, poor quality and heat-shocked bovine oocytes have higher *CTSB* activity than controls. Inhibiting *CTSB* activity increases the rate of development and improves embryo quality after in vitro fertilization (IVF) [23,24]. Taken together, the regulation of *CTSB* can serve as a promising tool to produce high-quality embryos in-vitro. These tidbits of evidence support the role of *CTSB* signaling by regulating folliculogenesis and embryogenesis.

Despite *CTSB*, expression is high in GC, and its functional role in folliculogenesis has not been elucidated. Herein, for the first time, we investigated the biological role of *CTSB* by employing small interference specific to *CTSB* in mice primary GC in vitro. Oocyte development and steroidogenesis were hypothesized to be controlled by *CTSB* in the GC. These processes were also examined in terms of the mechanisms by which the *CTSB* regulates them.

In this present study, we evaluated the silencing effects of *CTSB* on mouse primary GCs apoptosis, proliferation, cell cycle progression, and steroidogenesis with the underlying mechanism. Our results indicated that CTSB-KD suppressed apoptosis, autophagy, and steroidogenesis; increased proliferation; and enhanced the cell cycle progression through the AKT/ERK pathway by modulating *FSHR* and *CYP11A1*.

## 2. Results

### 2.1. siRNA Successfully Represses CTSB Expression

**Figure 1:**
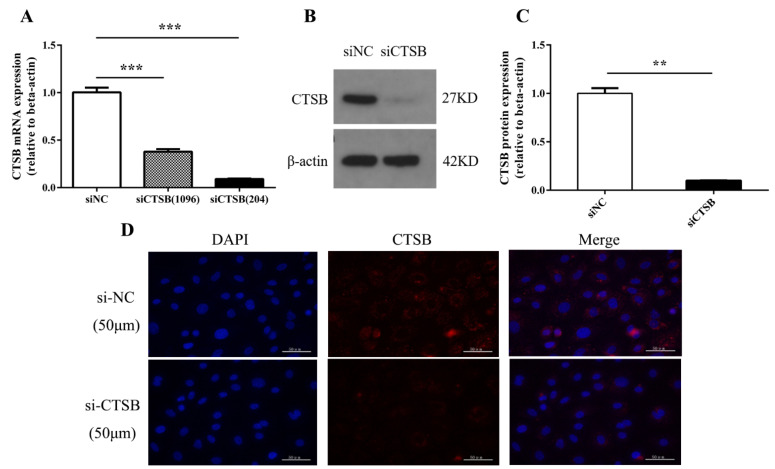
CTSB-KD inhibits *CTSB* expression in mouse GCs in vitro, both at the protein and mRNA levels. (**A**) CTSB mRNA expression was successfully decreased in mouse GCs transfected with siCTSB (100 nM). (**B**–**D**) GCs transfected with siCTSB (100 nM) exhibited a significant decrease in expression of CTSB at both the protein and mRNA levels in mouse GCs. Protein was determined by Western blotting and immunofluorescence, and mRNA levels by RT-qPCR relative to endogenous control β-actin. The results are indicated as means ± SEM of three independent experiments. ** *p* < 0.01, *** *p* < 0.001. siNC, negative control siRNA; *CTSB*, cathepsin B; GC, granulosa cells; siCTSB; and cathepsin B siRNA.

To uncover the biological function of *CTSB* in murine GCs, we designed and synthesized two different siRNAs: siCTSB (1096) and siCTSB (204). In brief, RT-qPCR, Western Blot, and immunofluorescence staining were used to determine siCTSB transcription and translation. The results shown in Figure 1 demonstrated that expression of *CTSB* is successfully inhibited in transfected murine GCs compared to control. The knockdown efficiency of *CTSB* mRNA level by siCTSB (204) transfection reached 91.16% (Figure 1A), compared to control siRNA-transfected cells. We selected siCTSB (204) for subsequent experiments because it was the most effective siRNA. Compared with the control group (1.00 ± 0.03), the *CTSB* protein relative expression of the siCTSB group (0.10 ± 0.01) was significantly lower (*p* < 0.001; Figure 1B,C).

### 2.2. CTSB Depletion Suppresses Apoptosis in Murine GCs In Vitro

**Figure 2:**
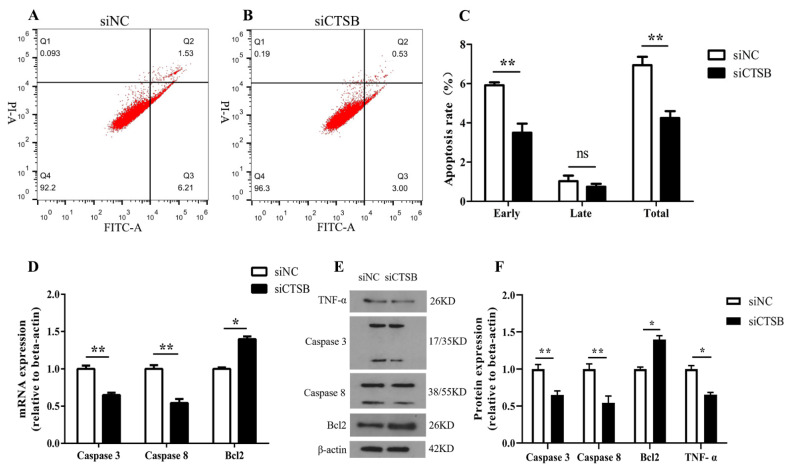
In vitro, *CTSB* KD inhibits apoptosis and suppresses the expression of apoptosis regulators in mouse GC. (**A**) (siNC) and (**B**) (siCTSB). Flow cytometry analysis was performed to detect dead cells in control and transfected cells (5 × 10^5^ cells per 6-well plate) using the annexin V-APC stain. (**C**) The rates of early, late, and total apoptosis were assessed. (**D**) In GCs transfected with siCTSB, the expression levels of apoptosis marker genes *caspase 3*, *caspase 8*, and *Bcl-2* were measured using RT-qPCR. (**E**,**F**). The expression levels of apoptosis marker genes (*caspase 3*, *caspase 8*, *Bcl-2*, and *TNF*) in siCTSB and NC-transfected GCs were determined using Western blotting against β-actin as an endogenous control. All results, at least from three independent experiments, are presented as means ± SEMs. * *p* < 0.05, ** *p* < 0.01. Early apoptosis = lower right (LR); late apoptosis = upper right (UR); viable cells = lower left (LL); necrotic cells = upper left (UL); FITC, v-fluorescein isothiocyanate; PI, propidium iodine; siNC, negative control.

The apoptosis rate was determined by transfecting murine GCs for 48 h with siCTSB or NC. Resulted showed that siCTSB-treated GCs had a significantly lower early apoptosis rate (3.50 ± 0.46) and total apoptosis rate (4.24 ± 0.36) compared to the control (early apoptosis rate, 5.91 ± 0.15; total apoptosis rate, 6.94 ± 0.42), respectively (*p* < 0.01; (Figure 2A–C).

Next, beneath molecular mechanism of murine GCs apoptosis mediated by *CTSB*, the expression level of apoptotic regulators (downstream) was examined, both at the mRNA and protein level. *Caspase 8* and *caspase 3* were inhibited in siCTSB-treated GC compared to control (*p* < 0.01), while *Bcl2* expression was promoted (*p* < 0.05). A significant reduction in *TNF* protein was also observed in the siCTSB group compared to the control groups (*p* < 0.05; Figure 2D–F). These results suggested that *CTSB* is involved in intrinsic apoptotic pathways in mouse GCs.

### 2.3. CTSB Downregulation Promotes Cell Proliferation and Affects Cell Cycle Progression in Mice GCs

**Figure 3:**
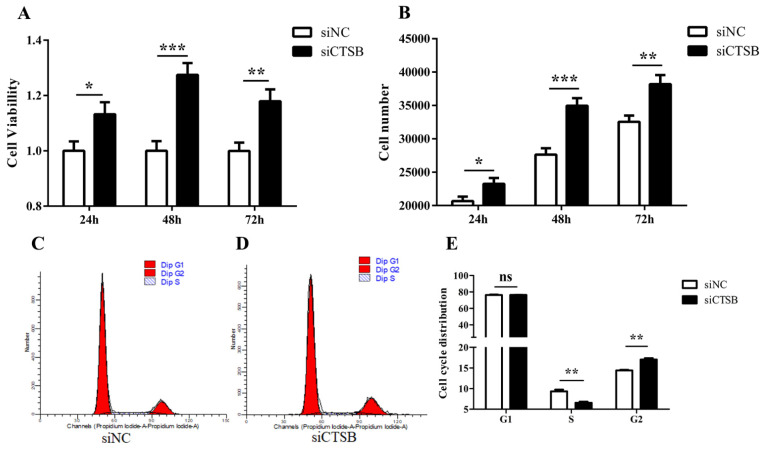
In vitro, *CTSB* depletion increased proliferation and modulated cell cycle progression in mouse GC. (**A**,**B**) The CCK-8 kit was used to assess the proliferation rate of GCs transfected with siCTSB and NC at 24, 48, and 72 h. The viability of the cells was determined by the concentration of formazan dye, a cell viability indicator. A multimode plate reader with absorbance at 450 nm was used to read the plates, and cell numbers were counted using an automated cell counter. (**C**–**E**) Mouse GC was transfected with siCTSB and incubated for 48 h before being saturated in PI, and the FACS and GC cycle were determined. All results are presented as means ± SEM. Significance difference, * *p* < 0.05, ** *p* < 0.01, *** *p* < 0.001; siCTSB, cathepsin B siRNA; GC, granulosa cells; siNC, negative control siRNA; ns, non-significant.

**Figure 4:**
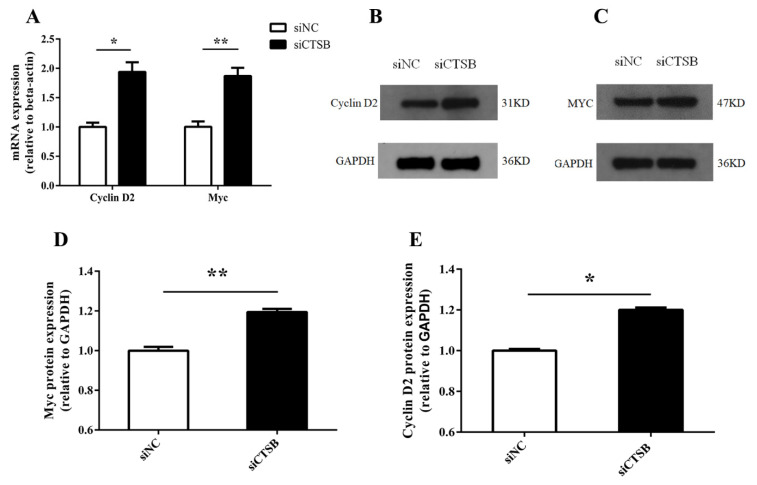
In vitro effect of *CTSB* depletion on expression levels (mRNA and protein) of proliferation and cell cycle marker genes in mouse GC. (**A**) Expression levels of *Myc* and *cyclin D2* genes in GC transfected with siRNA and NC were determined by RT-qPCR against endogenous control *β-actin*. (**B**–**E**) siCTSB promoted the protein expression level of *Myc* and *cyclin D2* in mouse GCs against an endogenous control *GAPDH*. The data were presented as means ± SEM of three independent experiments. Significant differences, * *p* < 0.05, ** *p* < 0.01; siCTSB, Cathepsin B siRNA; GC, granulosa cells; siNC, negative control siRNA.

After small interference-mediated downregulation of *CTSB* in murine GCS in vitro, GCs were evaluated for cell proliferation, cell number, and progression in the cell cycle. The CCk8 and cell-counting results of different time points (24, 48, and 72 h) showed that increase in GCs cell proliferation and cell number (0.11 ± 0.03 and 8200 ± 3166, respectively) was highly significantly (*p* < 0.01) at 48 h of transfection in si-CTSB-treated cells compared to that of the control (Figure 3A,B). Hence, we used the 48-h time point in the subsequent experiments. Flow cytometry analysis for cell cycle indicated the cell cycle was arrested in si-CTSB-treated cells compared to the control, as there was a significant decrease in S (2.78%) and increase in the G2 (2.63%) phase GCs (*p* < 0.01) (Figure 3C–E). We also examined the expression of downstream proliferation markers (*Myc* and *cyclin D2*) in mice to determine how *CTSB* controls GC proliferation. RT-qPCR and Western blot results shown in Figure 4 described a highly significant increase in *Myc* compared to its target gene, *cyclin D2*, at mRNA and protein expression levels (*p* < 0.01, and *p* < 0.05, respectively).

### 2.4. Downregulation of CTSB Mediates Mouse GC Proliferation by Activation of the ERK and Akt Phosphorylation Pathways

**Figure 5:**
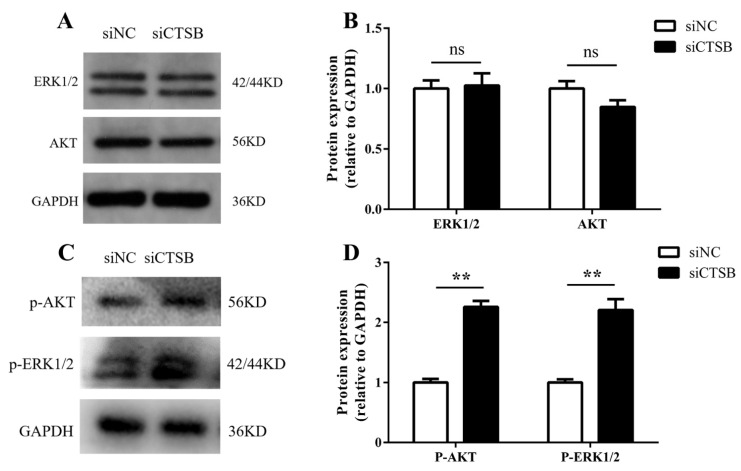
In vitro, *CTSB* downregulation activates ERK1/2 phosphorylation in mouse GCs. GCs transfected with siCTSB and siNC were harvested 48-h post-transfection for protein extracts. The level of total AKT/ERK 1/2 (**A**,**B**) and phosphorylated AKT/ERK 1/2 (**C**,**D**) proteins was quantified by Western blotting against endogenous control *GAPDH*. The data from three independent experiments were presented as means ± SEM, ** *p* < 0.01. siCTSB, cathepsin B siRNA; GCs, granulosa cells. siNC, negative control siRNA.

Following the *CTSB* footprints in GC proliferation, the pathways involved in CTSB’s functions in GC proliferation were investigated further. Western blotting was used to determine total and the phosphorylation levels of ERK1/2 and Akt serine–threonine kinase (Akt), downstream signaling pathways leading to cell proliferation. Total ERK1/2 expression between NC and siCTSB groups confirmed that expression of ERK1/2 was stable between two groups. In contrast to cells treated with siNC, CTSB-depleted cells had higher phosphorylation levels of Akt and ERK1/2 (*p* < 0.01; Figure 5). These results show that *CTSB* could be involved in regulating GC’s survival through ERK1/2 expression.

### 2.5. Steroidogenesis and Autophagy-Related Gene Expression Were Altered by CTSB Depletion in Mouse GCs In Vitro

**Figure 6:**
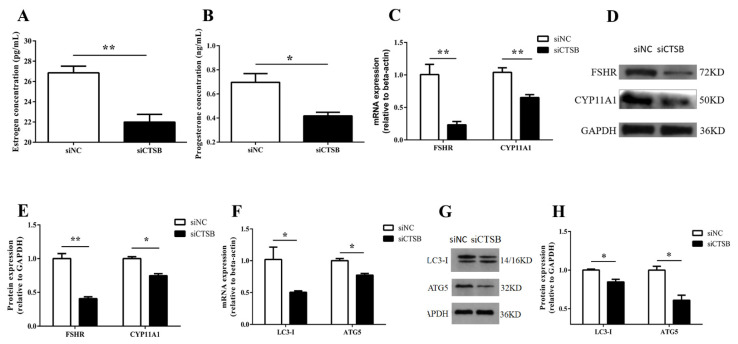
In vitro, *CTSB* deficiency altered the expression (mRNA and protein) of folliculogenesis and steroidogenesis marker genes in mouse GC. (**A**,**B**) At 48 h of GC transfection with siCTSB and NC, culture media was harvested, and estradiol and progesterone concentration was measured with an ELISA kit. (**C**–**E**) *FSHR* and *CYP11A1*, as well as (**F**–**H**) *LC3-I* and *ATG5*, were quantified at the mRNA and protein level through real-time PCR and Western blotting following siCTSB transfection. Results from three independent experiments were shown as means ± SEM. * *p* < 0.05, ** *p* < 0.01. siNC, negative control siRNA; siCTSB, cathepsin B siRNA; GCs, granulosa cells.

Whether knocking down of *CTSB* expression influenced the secretion of steroid hormones from GCs. To reveal the facts, at 48 h of transfection with siCTSB or NC, basal estradiol and progesterone production were detected in GCs culture media for steroidogenesis. As shown in Figure 6A,B, si-CTSB-treated GCs had a highly significant lower concentration of estradiol (26.86 ± 0.66 pg/mL), whereas the decrease in the progesterone (0.70 ± 0.07 ng/mL) concentration was significant lower compared to control (estradiol: 22.00 ± 0.755 pg/mL; progesterone: 0.42 ± 0.03 ng/mL) (*p* < 0.01, and *p* < 0.05), respectively. Mechanistically, a follicle-stimulating hormone receptor (*FSHR*), the cytochrome p450 family 11 subfamily A member 1 (*CYP11A1*), was downregulated both at mRNA, and protein expression levels in CTSB-depleted GCs (*p* < 0.01, and *p* < 0.05, respectively, Figure 6C–E). These results indicate that CTSB signaling regulates steroidogenesis via *FSHR* and *CYP11A1* and plays an important in oocyte development and intra-ovarian function. The higher progesterone estrogen ratio in culture media of transfected granulosa cells indicated that *CTSB* knockdown could promote GC luteinization. Further, we examined the mRNA expression of autophagy-related markers (*LC-3-I* and *ATG5*) to infer *CTSB* knockdown effects on autophagy. As shown in Figure 6F, the mRNA and protein expression level of *LC3-I* (0.50 ± 0.02) and *ATG5* (0.77 ± 0.026) were significantly decreased in the treatment group compared to the control (1.01 ± 0.19 vs. 1.00 ± 0.03, respectively). These results suggested that *CTSB* knockdown regulates steroidogenesis and autophagy through *FSHR*, *CYP11A1*, and *LC3-I*, *ATG5*, respectively.

## 3. Discussion

Despite *CTSB* potentially being involved in both normal and pathological functions, there are well-established roles in carcinogenesis (hepatocellular carcinomas [25], colon cancer [26], esophageal adenocarcinoma [27], pancreatic adenocarcinoma [28], cellular functions apoptosis [29], oxidative stress [30], and autophagy [31]). In different cells, *CTSB* was detected, including the bovine and porcine cumulus–oocyte complex (COCs) and embryos [20,21,22]. Despite mammalian ovary exhibited significant interplays and expression of *CTSB*, the knowledge about its biological functions and signaling related to female reproduction is still in its infancy. Interestingly, *CTSB* expression was found negatively correlated to bovine embryos quality. However, inhibition of *CTSB* activity decreased the developmental competency of preimplantation embryos both in bovine and porcine [23]. Consequently, *CTSB* was supposed to play a key role in GC functions, such as apoptosis, proliferation, cell cycle progression, and intra-ovarian functions. siRNA is widely used to knock down the target gene in any cell type with ease in delivery and convenience to use [32]. Using siRNA, the transcriptional and post-transcriptional abundance of *CTSB* was disrupted in mouse GCs to investigate its physiological function. In this experiment, siCTSB effectively suppressed *CTSB* expression at both the mRNA and protein levels (Figure 1).

Proliferation and apoptosis of GCs are essential physiological processes for cells [33,34]. A follicle’s fate is ultimately determined by the crosstalk between death and survival signals from GCs [2,35]. The endo/lysosomal compartment has been reported to cause cell death, particularly by cathepsin (Cts), which can regulate apoptosis [36]. Nevertheless, cellular context and Cts type determine Cts’ positive or negative influence on cell death [36,37,38,39]. It has been reported that Cts, B, H, L, and S can cleave classic caspase substrates, such as procaspase-1, -3, and -8 [40,41], and release proapoptotic mitochondrial enzymes *cytochrome c* [42,43], thus activating caspases and apoptosis. CTSB was reported to cause apoptosis through initiator caspases rather than executor caspases either directly or indirectly [18]. Previously, Guicciardi et al. found that *CTSB* might be involved in TNF-alpha-triggered apoptosis by releasing mitochondrial *cytochrome c* [43]. These studies concluded that *cathepsin B* has a vital role in TNF- α-induced apoptosis; however, exact mechanisms remain to be explored.

In this regard, the potency of *CTSB* in regulating mice GCs is not investigated. Findings of the current study described that *CTSB* downregulation suppressed apoptosis in mice GCs in vitro. In CTSB-depleted GCs, mRNA expression and protein level of apoptosis-related marker genes; caspase 8, an initiator caspase of extrinsic apoptotic pathway; and *caspase 3*, a key apoptosis executor among its family expression levels were reduced (Figure 2D–F). Furthermore, *CTSB* KD also decreased the expression of *TNF-α* in CTSB-depleted GCs (Figure 2E,F). *CTSB* mediated a decline in GCs apoptosis and marker genes, which is consistent with previous studies that suggest that CTSB signaling in mice GCs can activate apoptotic pathway mediated by apoptosis initiator *caspase 8* and concomitantly its interaction with apoptosis executioner *caspase 3*, thereby promoting apoptosis through extrinsic or caspase-8-mediated apoptosis. Previously, inhibition of *CTSB* was reported to reduce the apoptotic nuclei in the cumulus cell layer of IVM oocytes, probably due to the release of *CTSB* from lysosome into cumulus cells or oocytes in response to some stress, and thereby promoting apoptosis. Moreover, Xin Shan et al. reported that upregulation of *CTSB* expression had an association with upregulated apoptosis signaling pathway in atretic follicle granulosa cells (AFGCs) compared with healthy follicle granulosa cells (HFGCs) in porcine [44]. In this study, CTSB-depleted GCs from mice were shown to have reduced apoptosis for the first time, indicating that *CTSB* is a proapoptotic factor in immature cells.

CTSB-depleted granulosa cells were examined for proliferation in vitro. In this experimental study, different time points 24, 48, and 72, 48 h post-transfection, were slightly modified after being established in our lab to explore the GC proliferation [45], suggesting that the cells’ inherent biochemical and physiological properties depict cell proliferation. CTSB-depleted granulosa cells at 48 h had a significantly greater proliferation capacity and cell numbers than the NC group, suggesting that *CTSB* has antiproliferative behavior in mouse GC (Figure 3A,B). *CTSB* knockdown in mice GC increased G2/M cell numbers and decreased S-phase cell numbers, according to cell cycle assays (Figure 3C–E).

However, pieces of evidence have shown the diverse results of this study in some cancer cells. Li et al. found that knocking out *CTSB* inhibits cell proliferation in the human cholangiocarcinoma cell line QBC939 [46], and mice lacking *CTSB* had reduced cell proliferation in mammary carcinomas and their lung metastases. In cancerous tissues and cells, *CTSB* silencing inhibited proliferation. Despite not being precisely similar, these findings support our results due to survival effects of *CTSB* depletion.

Presently, the molecular mechanism to influence mouse GC proliferation mediated by *CTSB* signaling is not understood. Thereby, to further examine how *CTSB* regulates GC proliferation, our study showed that mRNA and protein expression levels of the proliferation-related genes, *Myc* and *cyclin D2*, were increased in response to *CTSB* depletion (Figure 4), reinforcing our proliferation assay results.

In gastric cancer cells, *Myc* upregulation was reported to increase the cells in the G2/M stage and decrease S-phase cells [47], agreeing with our findings. Opposite to the *cyclin D1* conflicting data, the *cyclin D2* (*CCND2*) gene is a recognized target gene of *Myc*. *Myc* interacts with *CCND2* promoter in humans through a single highly conserved E-box element in vivo [48,49]. It induces histone acetylation in a TRRAP-dependent manner and induces *CCND2* mRNA and protein expression [49]. Cell proliferation was retrained with inhibition of *CCND2* [50], and overexpression of *CCND2* induces cell proliferation [51]. Herein, depletion of *CTSB* increased *Myc* and *CCDN2* expressions; thereby, *Myc* promoted S/G2-phase cell cycle progression by upregulating the transcription of the *CCND2* gene.

*C-myc* is known as a positive regulator of *cyclin D2* transcription [48,49] and can be phosphorylated at Thr58 and Ser 62 by ERK1/2 [52,53]. ERK and AKT are essential in cell proliferation, death, and differentiation in almost all cell types [54]. AKT, also known as protein kinase B (PKB), mediates the phosphatidylinositol 3-kinase (PI3K) signaling pathway. The PI3K/AKT pathway regulates cell growth, proliferation, survival, motility, and invasion [55]. In addition to suppressing the functions of proapoptotic proteins, ERK1/2 can promote cell survival by enhancing the activity of anti-apoptotic molecules. Mcl-1, an anti-apoptotic member of the *Bcl-2* family, is phosphorylated at Thr163 by ERK1/2, thus increasing its stability and enhancing its anti-apoptotic activity [56]. Hence, we examined the AKT/ERK1/2 phosphorylation level in *CTSB* silencing GCs. The findings of the current study, which show the increase in the phosphorylation level of AKT/ERK1/2 (Figure 5C) and the increased expression of *BCl2* (Figure 2D,E) along with *c-myc* and *cyclin D2* (Figure 4A–D), infer that *CTSB* expression negatively regulates the proliferation of GC by AKT/ERK1/2 pathway.

Nevertheless, it remains to know if *CTSB* regulates steroidogenesis in GCs. In this study, we have demonstrated that *CTSB* downregulation induces a more significant decrease in E2 than P4 in GC culture media in vitro (Figure 6A,B). This finding suggests that *CTSB* could regulate the differential concentration of P4 and estradiol. For the first time, this study demonstrated that *CTSB* could regulate the secretion of E2 and P4 in GCs, and *FSHR* and *CYP11A1* may mediate its effects. As shown in Figure 6C–E, transcription and translation levels of *FSHR* and *CYP11A1* were significantly decreased in mice GCs treated with siCTSB. FSHR overexpression in granulosa cells was linked to the upregulation of proapoptotic genes and increased cell death than cells expressing relatively low *FSHR* levels [57]. Hence, low *FSHR* expression levels predominantly exhibited proliferative signals due to preferential activation of ERK signaling through B-arrestin. Altogether, these findings are consistent with the CTSB-KD-mediated low expression of *FSHR* and proliferative signals through ERK1/2 in the current study. However, further research is warranted to explore this detailed mechanism for *CTSB*.

The P450scc enzyme encoded by *CYP11A1* catalyzes the first and rate-limiting step in steroidogenesis by converting cholesterol to pregnenolone, a precursor to all other steroid hormones [58]. In other words, P4 biosynthesis is primarily dependent on *CYP11A1*; therefore, an aberrant expression of *CYP11A1* could fluctuate progesterone hormones levels [59]. *FSHR* expresses in ovarian granulosa cells to stimulate follicular maturation throughout the menstrual cycle and promote estradiol synthesis by aromatization [60]. The current study’s findings suggested that downregulation of *CTSB* could decrease the concentration of estradiol and P4 by reducing the expression of *FSHR* and *CYP11A1*, respectively.

The induction of autophagy in human granulosa cells was first described by Duerrschmidt et al. [11]. Some other studies in the PMSG rats model of follicular atresia have reported that autophagy in follicular atresia strongly correlates with apoptosis, as *LC3* and cleaved *caspase-3* expression was positively correlated with apoptosis [6]. Further, lysosomes are also involved in the degradation of regulators of steroidogenesis in the ovary, such as *LH–LHR* and *FSH–FSHR* complexes [61]. Taken together, considering the possibility of autophagy’s role in steroidogenesis in GCS, we examined the autophagy-related markers in *CTSB* silencing granulosa cells. The results revealed a significant decrease in mRNA expression of autophagy marker genes *LC3-1* and *ATG5*. A similar trend of decrease in *caspase 3* and *LC3-1* in CTSB-depleted GCs is consistent with previous studies, suggesting that *CTSB* might be regulating GCs through autophagy and apoptosis. CTSB-KD mediating a decrease in steroidogenesis, *CYP11A1*, and *ATG5* in GCs is also consistent with a previous study in mice which described a link between the deletion of the core autophagy gene *ATG5* as well as the reduced steroid levels and steroid deficiency phenotype mediated by *CYP11A1* [61,62]. However, further research in GCs from different stages follicles is warranted to explore the possible mechanism of *CTSB* in this context.

## 4. Materials and Methods

### 4.1. Management of Experimental Animals

This study was approved by the research approved by the Research Animal Ethics Committee of Huazhong Agricultural University (HZAUMO-2021–0016), and each experiment in this study was carried out in accordance with animal welfare standards. The experimental mice were purchased from Hubei Provincial Center for Disease Control. The mice were housed at Huazhong Agricultural University’s experimental animal center, where they had unlimited access to water and food and were subjected to a 12-h light/dark cycle.

### 4.2. Mouse GCs Isolation and Culture

The ovaries were collected from female KM (21 days old). Following an F12 (DMEM/F12) wash of pooled ovaries using needle puncture methods, GCs were isolated and purified three times using centrifugation (1000 rpm; 5 min) and DMEM/F12 wash. Pellets were saved after the supernatants were discarded. GCs were cultured in 6-well plates in culture media (DMEM/F12) with 10% FBS and 1% penicillin/streptomycin (Hyclone) and incubated for 48 h (37 °C; 5% CO_2_). During the incubation, culture media were replaced once at 24 h.

### 4.3. Cathepsin B (CTSB) siRNA Transfection

Both siRNA and NC-siRNA (without any target sequence) were purchased from Shanghai GenePharma Co., Ltd. (Shanghai, China). The cells were transfected with 100 nM of siRNA compared to NC using Lipofectamine RNAiMAX reagent in Opti-MEM medium (Life Technology, Inc., Carlsbad, CA, USA) instructions. Cells were harvested 48 h after transfection for mRNA and protein expression. Small RNA interference sequences specific to *CTSB* (siCTSB) used in this study are given in Table 1.

### 4.4. RNA Extraction and Reverse-Transcription Polymerase Chain Reaction (RT-PCR)

Cells were lysed 48 h after transfection to extract total RNA using a kit method (Total RNA Kit I (200); R6834-02; Omega Bio-Tek, Norcross, GA, USA) following the manufacturer’s instructions for subsequent cDNA synthesis. Spectrophotometry was used to determine the quality and quantity of RNA (Nanodrop 2000 Analyzer; Thermo Scientific, Wilmington, DE, USA). Finally, the kit method (FastKing RT Kit; TianGen Biotech, Co., Ltd., Beijing, China) was used for reverse transcription of 1 μg of qualified total RNA (260/280; 1.8–2.1), according to manufacturer instructions.

### 4.5. Quantitative Real-Time PCR (qRT-PCR)

Primers listed (Table 1) were designed using Primer 5.0 for this study. Gene expressions levels were determined by QRT-PCR (Light Cycler 480 Multiwell Plate 96; Roche, Indianapolis, IN, USA) using Bio-Rad (Bio-Rad, Inc., Hercules, CA, USA) against *β-actin* or *GAPDH* as an endogenous control. Each 10 μL of the reaction mixture was comprised cDNA (1 μL), RNase-free water (3 μL), sense and antisense primers (1 μL), and SYBR-Green Master Mix (QIAGEN, Hilden, Germany) (5 μL).

### 4.6. Protein Extraction and Western Blotting

GCs transfected with siCTSB and NC in a 6-well plate were placed on ice, washed with PBS, followed by cell lysis using a lysis buffer RIPA (Servicebio, Wuhan, China) augmented with phosphorylase inhibitor (1 mM) and phenylmethanesulphonyl fluoride (PMSF). Proteins were isolated by SDS-PAGE (12%) and subsequently transferred to polyvinylidene difluoride membranes (PVDF; Immobilon-P, Millipore) via electrophoresis. Preliminary, the membranes were incubated with 5% skimmed milk diluted in TBS for 2 h, and subsequently incubated (4 °C, overnight) with these primary antibodies: *Bcl2* (4223S), *CTSB* (31718), *cyclin D2* (3741P), p-ERK1/2 (4370S), *LC3-I* (2475S) (1:1000, CST, Danvers, MA, USA), *caspase 3* (A19654), *CYP11A1* (A1713), *FSHR* (A1480) (1:1000, Abclonal-Tech, Wuhan, China), *TNF-α* (17590-1-AP), *AKT* (10176-2-AP) (1:1000, Proteintech Group, lnc., Wuhan, China), *ERK* (1:1000, AF0155; Affinity Biologicals Inc. USA), p-Akt (Ab81283), *ATG5* (Ab109490) (1:2000; Abcam, Cambridge, UK); Abcam, Cambridge, UK), *MYC* (1:1000, NBP2-25147SS, Novus, CO, USA), *caspase 8* (1:1000, BS1387, Bioworld, Nanjing, China), *GAPDH* (1:1000, 60004-1-Ig; Proteintech Group, lnc. Wuhan, China), and *β-actin* (1:500, BM0627; BosterBio, Wuhan, China) as a control. Target proteins in each sample were determined using enhanced chemiluminescence (NCI5079; Bio-Rad, USA). WB-images were captured by Gel-Pro analyzer version 4 (Media Cybernetics, Rockville, MD, USA), and Image J software was used to determine bands, respectively. The data were normalized to*β-actin* or *GAPDH*.

### 4.7. Cell Apoptosis Detection

Flow cytometry was used to determine the apoptosis rate in mouse GCs cultured in 6-well plates 48 h after transfection with si-CTSB and NC using the FITC/ PI Apoptosis Detection Kit (KeyGEN Biotech, Nanjing, China) as directed by the manufacturer. The trypsin-lysed cells were washed twice with PBS before being resuspended in a binding buffer solution. The cells were stained (FITC and PI; 15 min, room temperature). Finally, flow cytometry was used to determine the apoptosis rate using the FACSVerse Calibur (BD Biosciences, San Jose, CA, USA) according to the manufacturer’s protocol. The apoptosis assay results were validated by examining the levels of mRNA and protein expression of apoptosis-related molecules.

### 4.8. Cell Counts and Proliferation Assay

Si-CTSB and NC-transfected GC were seeded in a 96-well plate and incubated for proliferation studies in a culture medium for 48 h. The cells that were trypsin-lysed were then counted using an automated cell counter (BIO-RAD Laboratories, Inc., TC20TM, Hercules, CA, USA). The kit method determined the capacity of GC proliferation (CCK-8; Dojindo Molecular Technologies, Inc., Rockville, MD, USA). As directed by the manufacturer, the viability of the cells was determined by the concentration of formazan dye, a cell viability indicator. In brief, each well-received 10% culture media with a 100-L CCK-8 solution was incubated for 3 h at 37 °C with 5% CO2 and saturated humidity. The GCs were then loaded onto a multimode plate reader (PerkinElmer, EnSpire, Waltham, MA, USA) to determine each experimental group’s absorbance at 450 nm.

### 4.9. Cellular Immunofluorescence

Using 4% paraformaldehyde in PHEM (0.5% Triton X-100), GCS were fixed for 45 min after PBS wash for immunofluorescence. Afterward, blocking was performed using PBS (2% BSA; 0.05% Tween-20, 1 h, Rm. Temp.). After that, GCs were incubated with a *CTSB* antibody (overnight at 4 °C) (1:800, CST, Danvers, MA, USA). Following three 10-min washes in PBS (0.05 percent Tween-20), GCs were incubated for one hour at 37 °C with Cy3-labeled goat anti-rabbit (Boster; 1:100) or TRITC-conjugated goat anti-human (Proteintech;1:50). PBS (1 g/mL DAPI; 10 min at room temperature) labeled the DNA. Finally, using DABCO, GC’s were mounted on glass slides for confocal microscopy (Zeiss LSM 510 META, Carl Zeiss Imaging, and Germany) equipped with a plan apochromat 63×/1.4 oil DIC objective. Zeiss LSM Image Browser and Adobe Photoshop were used to process the confocal images (Adobe Systems Inc., San Jose, CA, USA).

### 4.10. Cell Cycle Assay Middling

Following 48 h of transfection with si-CTSB and NC, mouse primary GCs were exposed to 0.25 percent trypsin (37 °C, 3 min) and centrifuged (4000 rpm, 5 min). Following a 70 percent ethanol wash and an overnight incubation at 4 °C, harvested cells were stained with RNase A and PI solution (100 μL and 400 μL, respectively; 30 min). Cell cycle progression was assessed by the FACSVerse Calibur (BD Biosciences, San Jose, CA, USA). ModFit was used to analyze the proportion of the cell cycle in different phases across three independent experiments.

### 4.11. Hormones Assay

Culture media were collected from si-CTSB and NC, transfected GCs at 48 h, and subjected to centrifugation (1000 g, 20 min) to ascertain hormone concentration. ELISA Kit (MLBIO Biotechnology Co., Ltd.; Shanghai, China) specific to mice was used to measure estradiol-17β (E2) and progesterone (P4) concentrations.

### 4.12. Statistical Analysis

Data indicated as mean ± SEM were obtained from three independent replicates at least. The statistically significant difference between groups was determined using paired-samples *t*-test in Graph-Pad. The cut-off value adjusted to a statistically significant difference was *p* < 0.05.

## 5. Conclusions

Our results established that siRNA-mediated downregulation of *CTSB* decreased the TNFa-mediated apoptosis and autophagy. Small interference-mediated downregulation of *CTSB* suppressed GC apoptosis; therefore, it can be concluded that *CTSB* serves as proapoptotic in mice ovarian cells. Furthermore, inhibiting *CTSB* promotes mouse GC proliferation via activation of the p-Akt and p-ERK1/2 pathways and significant changes in cell cycle progression. In this study, *CTSB* silencing induces a decrease in steroids hormones (progesterone and estradiol) secretion by suppressing the critical genes for steroid synthesis (*FSHR* and *CYP11A1*). Despite a novel role of *CTSB*, it is demonstrated herein that, other than recognized biological functions, additional experiments are warranted to confirm these findings in vivo.

Altogether, by illustrating the physiological role of the *CTSB* gene in an ovarian cell, this study suggests that future research should consider CTSB’s potential to improve fertility in female mammals and its use to develop new therapeutic approaches.

## Figures and Tables

**Table 1:** Primer sequences for quantitative real-time PCR and si-CTSB sequences.

Gene Name	AC/NO	Primer Sequences	Production Length (bp)	Annealing Temp. (°C)
*β-actin*	NM_007393	F: GTGACGTTGACATCCGTAAAGA	287	60
		R: GTAACAGTCCGCCTAGAAGCAC		
*Caspase 3*	NM_009810	F: GTCTGACTGGAAAGCCGAAAC	205	59.5
		R: GACTGGATGAACCACGACCC		
*Caspase 8*	NM_001080126	F: TCTCGGAATCGGTAGCAAACC	173	60
		R: AGAAGAGCTGTAACCTGTGGC		
*Cyclin D2*	NM_009829.3	F: TACCTCCCGCAGTGTTCCTA	158	60
		R: GCCAAGAAACGGTCCAGGTA		
*MYC*	NM_001177352.1	F: GTTGGAAACCCCGCAGACAG	264	60.5
		R: GTAGCGACCGCAACATAGGA		
*CYP11A1*	NM_001346787.1	F: TACTAACCTAGCCCGCCTCG	163	60.5
		R: CTCCTGCGCATAGAGAGAGC		
*FSHR*	NM_013523.3	F: AACACTTGCCAGCCTTTCAC	184	60
		R: TGGGTTCCGTTGAATGCACA		
*CTSB*	NM_007798.3	F: CAATGGCCGAGTCAACGTG	176	59
		R: TGGTGTATGGTAAGCAGCCT		
*BCL2*	NM_009741.5	F: GAACTGGGGGAGGATTGTGG	194	60
		R: GCATGCTGGGGCCATATAGT		
*LC3-I*	NM_025735.3	F:AGGAGAAGGATGAAGACGGA	160	57
		R:CCACTGGGGACTGAAATAGC		
*ATG5*	NM_001358596.1	F:CAGTGTGATCCCGGCAGA	199	58
		R:GAGTAAAGCAAGTTGGAATTCG		
si-CTSB (204)		F: GCUGUCGGAUGACCUGAUUTT		
		R: AAUCAGGUCAUCCGACAGCTT		
si-CTSB (1096)		F: UCAGAAAUUGUGGCUGGAATT		
		R: UUCCAGCCACAAUUUCUGATT		

## Data Availability

The authors confirm that the data supporting the findings of this study are available within the article.
